# Validity of Consumer Activity Monitors and an Algorithm Using Smartphone Data for Measuring Steps during Different Activity Types

**DOI:** 10.3390/ijerph17249314

**Published:** 2020-12-12

**Authors:** Verena Hartung, Mustafa Sarshar, Viktoria Karle, Layal Shammas, Asarnusch Rashid, Paul Roullier, Caroline Eilers, Mathias Mäurer, Peter Flachenecker, Klaus Pfeifer, Alexander Tallner

**Affiliations:** 1 Department of Sport Science and Sport, Friedrich-Alexander University Erlangen-Nürnberg, 91058 Erlangen, Germany; klaus.pfeifer@fau.de (K.P.); alexander.tallner@fau.de (A.T.); 2 Department of Sport Science, Division of Health and Physical Activity, Otto-von-Guericke University, 39104 Magdeburg, Germany; mustafa.sarshar@ovgu.de; 3 Department of Education, University of Regensburg, 93040 Regensburg, Germany; viktoria.karle@ur.de; 4 Zentrum für Telemedizin Bad Kissingen, 97688 Bad Kissingen, Germany; shammas@ztm.de (L.S.); rashid@ztm.de (A.R.); roullier@ztm.de (P.R.); 5 Department of Neurology, Klinikum Würzburg Mitte gGmbH, 97070 Würzburg, Germany; c.eilers@kwm-klinikum.de (C.E.); m.maeurer@kwm-klinikum.de (M.M.); 6 Neurological Rehabilitation Center Quellenhof, 75323 Bad Wildbad, Germany; peter.flachenecker@sana.de

**Keywords:** accelerometer, accuracy, activity trackers, smartphone, validation study, walking, activities of daily living

## Abstract

*Background*: Consumer activity monitors and smartphones have gained relevance for the assessment and promotion of physical activity. The aim of this study was to determine the concurrent validity of various consumer activity monitor models and smartphone models for measuring steps. *Methods*: Participants completed three activity protocols: (1) overground walking with three different speeds (comfortable, slow, fast), (2) activities of daily living (ADLs) focusing on arm movements, and (3) intermittent walking. Participants wore 11 activity monitors (wrist: 8; hip: 2; ankle: 1) and four smartphones (hip: 3; calf: 1). Observed steps served as the criterion measure. The mean average percentage error (MAPE) was calculated for each device and protocol. *Results*: Eighteen healthy adults participated in the study (age: 28.8 ± 4.9 years). MAPEs ranged from 0.3–38.2% during overground walking, 48.2–861.2% during ADLs, and 11.2–47.3% during intermittent walking. Wrist-worn activity monitors tended to misclassify arm movements as steps. Smartphone data collected at the hip, analyzed with a separate algorithm, performed either equally or even superiorly to the research-grade ActiGraph. *Conclusion*: This study highlights the potential of smartphones for physical activity measurement. Measurement inaccuracies during intermittent walking and arm movements should be considered when interpreting study results and choosing activity monitors for evaluation purposes.

## 1. Introduction

The reduction of physical inactivity is a global health goal [1]. Physical inactivity is associated with premature death and a number of chronic diseases like breast and colon cancer, diabetes mellitus, and coronary heart disease [2]. However, about 36.8% of the population in high-income countries remain inactive [3]. Against this background, the measurement of physical activity (PA) plays an important role in health promotion [4]. It is, among others, used for PA surveillance, the evaluation of PA promotion programs [4], and for the provision of feedback in behavior change programs [5,6]. Numerous subjective (e.g., diaries and questionnaires) and objective methods (e.g., doubly labeled water, indirect calorimetry, and accelerometry) exist that can be used to determine PA. These methods all have specific advantages and disadvantages [7].

The objective measurement of PA is often conducted with activity monitors [8]. Activity monitors can be defined as “devices used to conduct physical activity measurement” [6] (p. R359). These monitors often use accelerometers that process acceleration signals to derive PA metrics like steps, type, or intensity of PA [9]. Additional built-in sensors like gyroscopes, inclination, light, or heart rate sensors may improve the accuracy of PA measurement [10]. However, activity monitors that have been used in research studies for measuring PA have shortcomings compared to questionnaires and pedometers [7]. Data transfer and processing has to be done by scientific staff and is relatively time consuming. Furthermore, devices are rather expensive and usually do not provide real-time feedback [7].

In recent years, commercially available activity monitors have gained popularity on the consumer market with a growing number of new devices being released each year [11]. Along with this development, their use in research studies has increased [6,12]. Consumer activity monitors (CAMs) have been used to inform treatment decisions in medical settings and as a feedback tool to promote behavior change [12]. CAMs are mostly wrist-worn and equipped with triaxial accelerometers [13]. Types of CAMs are fitness trackers, smartwatches [14], and hybrid watches [11]. Compared to research-grade accelerometers, CAMs offer the possibility for continuous PA measurement over a period of months or years [6], are easy to synchronize with a smartphone or computer application [15], and contain behavior change techniques [15,16].

Next to CAMs, smartphones gain relevance for PA measurement as well [17]. The Pew Research Center [18] estimates that about 76% of people living in advanced economies own a smartphone. If validity and sufficient wear time can be assured, the use of smartphones for PA tracking would make additional measurement devices redundant [17]. In addition, smartphones have the advantage that raw sensor data is freely accessible and can be used to feed research-based, step-detection algorithms.

When CAMs and smartphones are used for the measurement and promotion of free-living PA, the validity of provided PA metrics is of utmost importance [6,17]. Here, the accurate identification of steps is specifically important. Steps are not only a common measure of PA that is associated with various health outcomes [19,20], they are also often used to derive other PA metrics (e.g., distance) [21]. A plethora of studies has investigated the validity of different CAMs for measuring steps under laboratory conditions using standardized continuous walking protocols [22,23,24,25]. In these studies, observed steps usually served as the criterion measure. This direct observation is very difficult to realize under free-living conditions. To date, only one study has succeeded in genuinely observing free-living PA by attaching a camera to participants’ torsos to record steps [26]. In this study, CAMs have consistently underestimated actual free-living steps. Thus, assessing validity of activity monitors with standardized continuous walking protocols does not necessarily reflect validity for free-living walking conditions. To investigate potential sources of error, some studies measured step count accuracy during arm movements, activities of daily living (ADLs), and intermittent walking [27,28,29,30]. While step count accuracy was high for some activities, it was rather low for other activities [27,28,29,30]. This indicates a need for further studies investigating the step-detection accuracy during different types of activities. These studies can increase our understanding of sources of measurement error during free-living PA. Furthermore, few studies exist that determine the validity of current CAM models for measuring steps. Those studies investigate the validity during continuous walking in standardized settings and free-living conditions—e.g., [17,31]. To our knowledge, no study investigated the validity during arm movements, ADLs, or intermittent walking. However, studies investigating the validity of current CAM models for measuring steps are needed, since results for outdated models cannot be transferred automatically to new models [6]. To sum up, CAMs and smartphones are promising PA measurement tools. Their validity for measuring steps should be analyzed not only for continuous walking in the laboratory setting but also for activities focusing on arm movements and intermittent walking. In addition, little is known about the validity of current CAM models for measuring steps.

Thus, the aim of this study was to determine concurrent validity of (1) eight different recently released CAMs and (2) an algorithm using smartphone data for measuring steps during three different types of activities: overground walking, ADLs focusing on arm movements, and short intermittent walking. Additionally, one research-grade activity monitor was analyzed as a reference for step-detection accuracy.

## 2. Materials and Methods

This study was part of the “MS bewegt” project that aimed at developing and evaluating an internet-based exercise and PA promotion intervention for persons with multiple sclerosis (PwMS) (NCT04367389). To inform subsequent studies evaluating the validity of CAMs and smartphones for measuring steps in PwMS, this pilot study was conducted in healthy adults.

### 2.1. Participants

Participants were recruited from the Department of Sport Science and Sport of the Friedrich-Alexander University Erlangen-Nürnberg (FAU) through mailing lists, leaflets, and word of mouth. Thus, potential participants were current or former students as well as employees at the Department of Sport Science and Sport. Healthy participants with a minimum of 18 years of age were included. Exclusion criteria were mental or physical difficulties, which may affect walking or arm movements. Study staff screened possible participants for eligibility through interviews. Eligible participants provided informed, written consent prior to participation. This study was approved by the ethics committee of the Baden-Württemberg Federal Chamber of Physicians (F-2018-024).

### 2.2. Procedures

Data were collected in July and August 2019. First, participants self-reported sociodemographic information including age, sex, height, and body mass as well as dominant foot and hand. Second, participants were equipped with 10 CAMs, the research-grade activity monitor ActiGraph wGT3X-BT (ActiGraph LLC, Pensacola, FL, USA), and four smartphones. Attachment sites were either the wrist, hip, calf, or ankle. Specifications of the devices as well as an overview of the attachment sites applied in this study are shown in Table 1.

**Figure 1:**
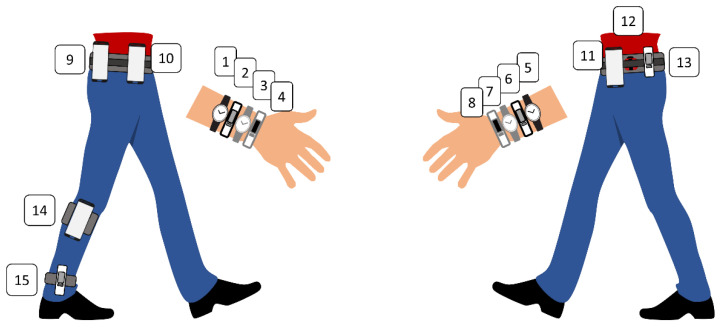
Sample constellation displaying all 15 wear positions.

A recent review demonstrates that the ActiGraph is one of the most commonly used research-grade activity monitors in PA promotion studies [8]. Therefore, the ActiGraph was included as a reference for step-detection accuracy of smartphones and CAMs. Fitness trackers, smartwatches, hybrid watches, and smartphones were chosen from a variety of popular brands and price ranges. Wear positions for activity monitors and smartphones were standardized (Figure 1). Two narrow and two wide CAMs were worn alternatingly on each wrist, totaling eight wrist-worn devices. To exclude systematic bias, wear positions were permuted after each participant, ensuring that each device was worn once at each of the eight positions after eight participants. The wear position was randomized for each participant beforehand. Five devices were worn on the hip, attached to an elastic band. The ActiGraph was constantly worn on the left hip, above the anterior superior iliac spine. Three smartphones (Samsung Galaxy S10e—Samsung, Seoul, South Korea, Sony Xperia—Sony Corporation, Tokyo, Japan, and Nokia 8—Nokia Corporation, Espoo, Finland) and one Fitbit Inspire (Fitbit Inc., San Francisco, CA, USA) were worn on four positions: left anterior, right anterior, left posterior, and right posterior. Those wear positions were also randomized for each participant beforehand and permuted after each participant to ensure that each device was worn once at each of the four positions after four participants. Smartphones were placed in vertical orientation within a firm case at the most lateral position possible that did not hamper arm swing during walking. The Fitbit Inspire was positioned 2 cm medial of the anterior superior iliac spine or on the posterior superior iliac spine. Additionally, one smartphone (Nokia 8) was positioned with an elastic band on the lateral side of the right calf and one Fitbit Inspire on the lateral side of the right ankle.

Three structured activity protocols were completed in the following order: overground walking, ADLs focusing on arm movements, and intermittent walking. Steps were recorded with a hand tally counter. This served as the criterion measure. A step was defined as a forward, sideward, or backward displacement of the foot together with a forward displacement of the trunk. Closing steps were not counted.

The overground walking protocol consisted of three separate tests. In each test, participants were asked to complete 250 steps on a 30-m corridor with turning points. The first test was conducted at participants’ comfortable walking speed. In the second and third tests, participants were asked to walk 20% faster and 20% slower, respectively. The order of the second and third tests was randomized. The first assessor (V.H., M.S., or A.T.) walked behind the participant and measured walking distance and speed with a measuring wheel that was equipped with a speedometer. The second assessor (V.H., M.S., V.K., or A.T.) counted steps and recorded the time needed to walk 250 steps. The average comfortable speed from the first test was computed and used to determine the target walking speeds for the second and third tests. The first assessor informed the participants during those tests to walk slower or faster, if needed. At the turning points, participants were told to walk an arc without altering their walking speed. To facilitate stopping after 250 steps, the last five steps of each test were counted down.

During the second protocol, participants completed a sequence of ADLs focusing on arm movements including washing one’s hands, washing dishes, stacking plates, and simulating eating behavior. These activities were chosen to estimate the number of false positive steps that may be counted during everyday activities. The test setup was developed to reduce walking for transitioning between different activities to a minimum. A detailed protocol description including participant instructions and test setup is provided in Appendix A. Overall, the test included 40 repetitive upper extremity movements plus nonrepetitive movements during hand and dish washing.

**Figure 2:**
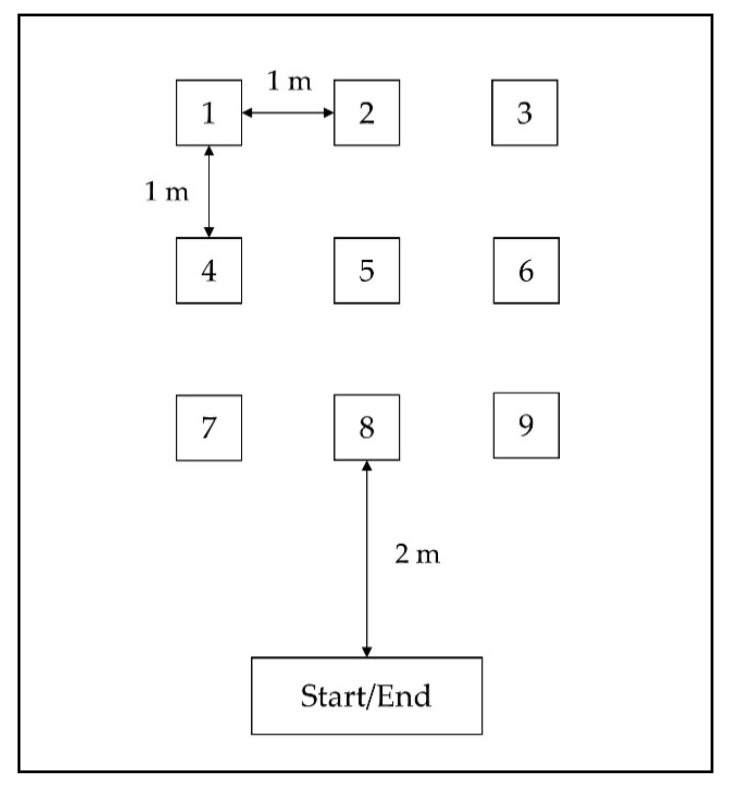
Test setup for the intermittent walking activity protocol.

Lastly, participants completed a protocol with short, intermittent walking bouts. Short, intermittent walking bouts are a potential source of underestimation during free-living PA. Methods applied by manufacturers to reduce false positive steps during nonwalking behavior may also eliminate steps during intermittent walking [30]. For this protocol, nine numbered marks arranged like numbers on a keyboard and a mark for the starting point were attached to the floor (Figure 2). Participants were asked to walk from the starting point to these nine positions in an ascending order. When one numbered mark was reached, participants were instructed to stand still for one second, then turn and return to the starting point. Participants completed the parkour twice and ended the protocol at the starting point, totaling 36 short walking bouts.

Prior to the ADL and the intermittent walking protocol, the first assessor explained the protocol to the participant. During both tests, the first assessor gave verbal cues to assist the participant and the second assessor counted steps with a hand tally counter. To determine the accuracy of step counting with the hand tally counter, steps were recorded by two out of four researchers (V.H., V.K., M.S., and A.T.) for three participants.

### 2.3. Data Collection and Analysis

Before and after each test, participants were asked to stand still for about 1–2 min to allow the assessors to start and stop data recording on the smartphones, set a marker for the ActiGraph (intensive shaking), and read the number of steps from the displays of CAMs.

Raw acceleration data from smartphones were collected with the smartphone application Phyphox, developed by the RWTH Aachen University (RWTH Aachen University, Aachen, Germany) [32]. Smartphones’ raw acceleration signals were analyzed with a step-detection algorithm using zero-crossing detection developed by the Zentrum für Telemedizin Bad Kissingen (Zentrum für Telemedizin Bad Kissingen, Bad Kissingen, Germany) [33]. This algorithm had already demonstrated high sensitivity (99.95%) during treadmill and overground walking [33]. Acceleration signals were processed using Matlab (The MathWorks, Inc., Natick, MA, USA). ActiGraph data were collected at 100 Hz and downloaded using the software ActiLife (version 6.13.4; ActiGraph LLC, Pensacola, FL, USA). Data were converted to 1-s epochs using the low frequency extension filter. Steps and counts for the three axes were calculated for each second using the ActiLife software. Based on this, steps were determined for each test. Furthermore, the time needed to complete the ADL and the intermittent walking protocol was determined based on the ActiGraph data.

Statistical analysis was performed with IBM SPSS Statistics 25 (IBM, Armonk, NY, USA). Inter-rater agreement for each activity protocol was determined by calculating the percentage of observations with a difference between assessors of zero step and one step or fewer [34]. Univariate outliers were identified by calculating z-scores for each observation. Outliers were defined as having a z-score of 2.59 or greater [35]. Mean number of steps and standard deviation (SD) were calculated for all devices and activity protocols. To establish concurrent validity of devices for measuring steps, the mean difference in steps (± SD) (step count activity monitor—actual step count) and the mean absolute percentage error (MAPE) were calculated for each activity protocol and device. MAPE was calculated as follows:

(1) $$\left(\frac{1}{n}\overset{n}{\underset{i\;=\;1}{\sum }}\left|\frac{{\mathrm{step}\;\mathrm{count}\;\mathrm{activity}\;\mathrm{monitor}}_{i}\;-\;{\mathrm{actual}\;\mathrm{step}\;\mathrm{count}}_{i}}{{\mathrm{actual}\;\mathrm{step}\;\mathrm{count}}_{i}}\right|\right)\times 100\%$$

In this equation, *n* represents the number of trials analyzed. In accordance with previous studies [36,37,38], a MAPE < 3% was considered acceptable for overground walking. Graphs of mean steps and SDs were created using Python (version 3.7; Python Software Foundation, Wilmington, DE, USA).

## 3. Results

Eighteen participants were included in the study (Table 2). Data from one overground walking trial were excluded prior to analysis, since the number of observed steps was missing. Missing data per variable and test were low (0.0–5.6%). For the overground walking test, 0–3 outliers were identified per device (0 outliers: Nokia 8—calf; 1 outlier: Samsung Galaxy Watch Active; 2 outliers: Fitbit Inspire, Fitbit Ionic, Garmin vivovit, Withings Pulse, Withings Steel, Fitbit Inspire—ankle, ActiGraph; 3 outliers: Garmin vivomove, Mi Band, Fitbit Inspire—hip, Samsung Galaxy S10, Sony Xperia, Nokia 8—hip). During the ADLs only one observation (Samsung Galaxy S10) was identified as an outlier. Three devices produced one outlier in the intermittent walking protocol (Fitbit Inspire—ankle, Samsung Galaxy S10, Nokia 8—calf). All available data were used for analysis. Outliers were retained in the analysis, since it was assumed that they represent valid observations. When two assessors counted steps, 55.6% of observations during overground walking differed by zero step and 88.9% of observations differed by one step or fewer. Steps counted by two assessors during the ADL protocol differed by zero step in 0.0% of observations and by one step or fewer in 100% of observations. During the intermittent walking protocol two assessors differed by zero step in 66.7% of observations and by one step or fewer in 100% of observations.

**Figure 3:**
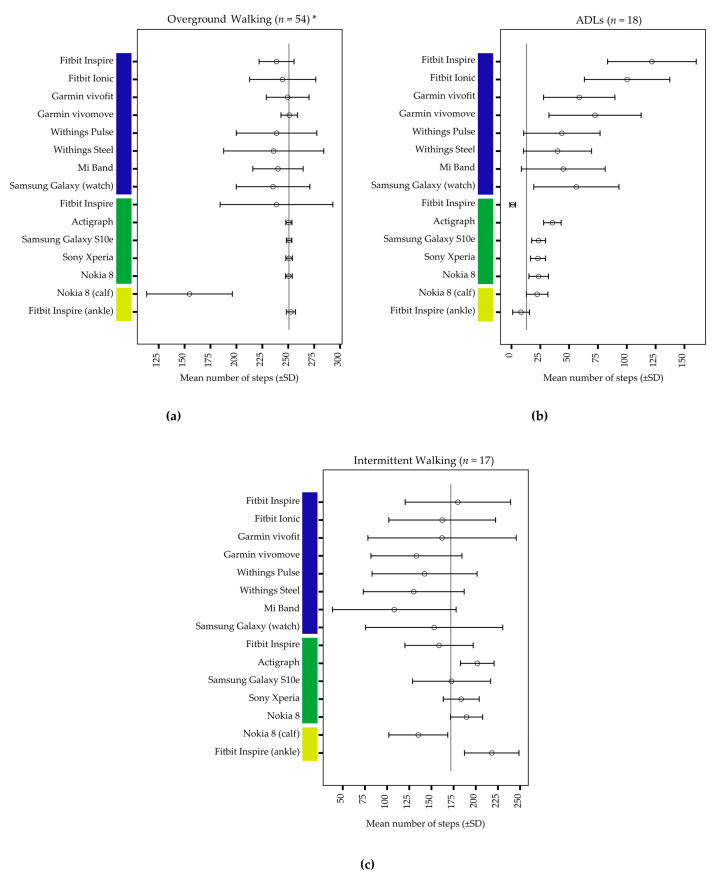
Mean values and standard deviation (SD) of all devices for (**a**) overground walking, (**b**) activities of daily living (ADLs), and (**c**) intermittent walking. Average observed steps are shown as a vertical, grey line. The blue bar identifies devices worn on the wrist, the green bar devices worn on the hip, and the yellow bar devices worn on the calf or ankle. * Each of the 18 participants completed three overground walking tests.

Figure 3 shows means (SD) of steps detected for each activity protocol and device. Mean differences (SD) as well as MAPEs for all activity protocols and devices are shown in Table 3.

### 3.1. Overground Walking

The mean actual steps were 250.7 (SD = 1.8) for overground walking. Participants walked on average with a speed of 4.9 km/h (SD = 0.9, 95% CI—4.6, 5.1) for 135 sec (SD = 15, 95% CI—131.5, 139.5). The average absolute difference between calculated target speed and actual speed for the slow and the fast walking trial was 0.16 km/h (SD = 0.13). During overground walking seven out of 15 devices had a MAPE < 3%: the hip-worn smartphones (Samsung Galaxy S10e, Sony Xperia, Nokia 8) and the ankle-worn Fitbit Inspire, the ActiGraph, the Garmin vivofit, and the Garmin vivomove. The Nokia 8 worn on the calf was the least accurate (MAPE = 38.2%). Most devices tended to underestimate actual steps.

### 3.2. Activities of Daily Living

Participants walked on average 13.2 (SD = 3.0) steps between the different types of ADLs as measured with the hand tally counter. The average time needed to complete the test protocol was 215 sec (SD = 23, 95% CI—203.7, 226.8). Smartphones as well as hip- and ankle-worn activity monitors were more accurate with MAPEs between 48.2% (ankle-worn Fitbit Inspire) and 177.3% (ActiGraph) than wrist-worn CAMs with MAPEs between 208.6% (Withings Steel) and 861.2% (Fitbit Inspire). All devices, except for the hip- and ankle-worn Fitbit Inspire, tended to overestimate steps.

### 3.3. Intermittent Walking

Participants walked on average 171.9 (SD = 13.6) steps within an average of 170 sec (SD = 23, 95% CI—158.5, 182.3). The hip-worn smartphones measured steps most accurately with MAPEs ranging between 11.2% and 12.5%, followed by the ActiGraph (MAPE = 17.8%) and the hip-worn Fitbit Inspire (MAPE = 19.3%). The Garmin vivofit, the Mi Band, and the Samsung Galaxy Watch were least accurate. While seven of eight wrist-worn activity monitors and the Nokia 8 on the calf tended to underestimate steps, three of four hip-worn devices and the ankle-worn Fitbit had a tendency to overestimate steps.

## 4. Discussion

The accuracy of tested devices was strongly dependent on the type of activity protocol. The lowest measurement errors were observed during overground walking, followed by intermittent walking and ADLs. This is in accordance with other studies that found smaller errors during continuous walking as compared to ADLs and arm movements while sitting [27,29].

### 4.1. Overground Walking

During overground walking, only the wrist-worn Garmin devices, the ankle-worn Fitbit Inspire, the hip-worn smartphones, and the ActiGraph achieved a MAPE below 3%. Thus, six of eight wrist-worn devices could not fulfill the predetermined quality criteria, compared to only one of four hip-worn devices, the Fitbit Inspire. In earlier studies, hip-worn Fitbit models (Fitbit One and Fitbit Zip) outperformed wrist-worn devices during overground walking with higher measurement accuracies than in our study [39,40]. The higher error in our study is largely attributable to results from one participant. For this person, the hip-worn Fitbit Inspire consistently identified 17 to 18 steps during the three overground walking trials. A previous study using the hip-worn Fitbit Ultra identified individual participants that had walking minutes with zero step counts [41]. The authors argue that while some factors that can influence step count accuracy are already known, such as device positioning, inclined walking, or walking speeds below a self-selected pace, further studies are necessary to increase understanding of such inaccuracies [41].

The ZTM algorithm using data from smartphones attached to the hip (Samsung Galaxy S10e, Sony Xperia, Nokia 8) calculated steps very accurately with MAPEs from 0.3–0.6%. Presset et al. [42] analyzed step count accuracy of the Runtastic Pedometer smartphone application during treadmill walking for data collected at the hip. They reported MAPEs of 19.3% (2 km/h), 1.5% (4 km/h), and 0.7% (6 km/h). Beltrán-Carrillo et al. [43] reported relative biases of −28.1% and 5.4% for the Samsung Health App and different hip-worn smartphone models during overground walking. Thus, the ZTM step-detection algorithm seems to outperform those consumer smartphone applications when data are collected at the hip. Furthermore, the accuracy of the smartphone data analysis was comparable to the accuracy of the research-grade activity monitor ActiGraph (MAPE = 0.5%). However, the Nokia 8 attached to the calf did not measure steps accurately with a MAPE during overground walking of approximately 40%. A reason for this high error might be that the ZTM algorithm was specifically developed for acceleration data collected at the hip.

### 4.2. Activities of Daily Living

During ADLs, wrist-worn devices considerably overestimated steps with MAPEs from 200–860% and were less accurate than hip- and ankle-worn devices. Other studies investigating step-detection accuracy during ADLs and specific upper extremity movements show heterogeneous results [27,28,29]. Nelson et al. [29] investigated the accuracy of the Fitbit One, Zip and Flex, the Jawbone UP, and the Omron pedometer during sedentary activities including arm movements (e.g., playing cards, writing, watching television, reading). Wrist-worn devices overestimated steps during sedentary activities, counting on average 1–2 steps within 10 min. The hip-worn Fitbit Zip and Fitbit One did not measure any steps. Alinia et al. [28] measured the accuracy of the wrist-worn Fitbit Flex during eating an apple while sitting. The activity monitor counted 4.8 steps per minute. Chen et al. [27] measured steps while participants played a tablet game or folded laundry for 5 min in a sitting position. Wrist-worn devices (Jawbone Up, Fitbit Flex, Garmin vivofit) counted between 0.0 and 2.1 steps during playing a tablet game. While folding laundry, those devices counted between approximately 90 and 180 steps. Results from these studies show that the amount of overestimation varies between devices and movements. Projected to a time span of 10 min, the overestimation of wrist-worn devices in these studies ranges between 1 (sedentary activities) [29], 48 (eating an apple) [28], and 180–360 (folding laundry) steps [27]. In our study, the overestimation of wrist-worn devices within 10 min was approximately between 75 (Withings Steel) and 300 (Fitbit Inspire) steps. Reasons for differences among studies may be caused by the different testing protocols. The activities used in our protocol were highly standardized and repetitive, in sets of 10 consecutive, uniform movements. This pattern was deliberately chosen to mimic the repetitive character of walking. This upper extremity movement pattern was considered suitable to detect susceptibility of devices to count false positive steps but may not exactly reflect free-living situations. Furthermore, as Chen et al. [27] already concluded, continuous, large, arm movements are more likely counted as steps. This may have contributed to differences observed in previously mentioned studies and high error rates in our study.

In line with the aforementioned studies, our results showed that devices worn at the hip or ankle are considerably less prone to record false positive steps during arm movements. This includes steps derived from smartphone data collected at the hip. The ZTM algorithm had an even lower MAPE in any of the hip-worn smartphone models than the ActiGraph. This speaks for the suitability of smartphones for valid assessment of free-living PA.

### 4.3. Intermittent Walking

During intermittent walking, there were tendencies for underestimation as well as overestimation, probably caused by data filters in consumer devices and the applied step count definition.

Most CAMs tended to underestimate steps with MAPEs from 28% to 47%. This is in line with findings from a previous study on intermittent walking [30]. Toth et al. [30] showed that CAMs underestimate steps during walking bouts of four to 12 steps. Furthermore, a walking bout was broken up into two bouts after 1–2 s of rest by all CAMs.

Hip-worn smartphones, the ActiGraph, and the ankle-worn Fitbit tended to overestimate steps during intermittent walking in our study. Reason for this may be the step definition applied to count steps manually and the absence of obvious methods to reduced false positive steps in case of the ActiGraph [30] and the ZTM algorithm [33]. In our study, researchers did not count the closing step leading to a parallel stance at the markers as a step. Despite presumably lower acceleration signals compared to normal walking steps, signal intensity of closing steps may have been sufficient to trigger step detection in devices worn at the hip or ankle. This seems to hold true especially for acceleration at the ankle, as the ankle-worn Fitbit Inspire was the device with the highest overestimation of steps. According to the protocol, participants had 36 closing steps in total, which comes close to the mean overestimation of 46 steps measured at the ankle.

To our knowledge only one study examined step-detection accuracy and precision during intermittent walking for a consumer smartphone application [44]. Attachment site of the smartphones was the sacrum. In this study, accuracy of step detection was dependent upon the number of continuous steps walked, with a minimum of five continuous steps needed for an accuracy exceeding 60%. In our intermittent walking protocol, the section lengths were about 2 m, 3 m, and 4 m. Despite these short distances, the ZTM algorithm did not underestimate steps and yielded a more accurate estimation of steps than the smartphone application used in the study from Brodie et al. [44].

### 4.4. Observations on Device Level

Over all activity protocols, hip-worn and ankle-worn devices seemed to outperform wrist-worn devices. Among the CAMs, the Fitbit Inspire worn at the ankle produced the best results in all tests, taking into account that most of the measurement error during intermittent walking may be attributable to closing steps that were not counted by assessors. Among the wrist-worn devices, different models from one manufacturer seemed to share strengths or weaknesses. The two Garmin models were the only devices to show a MAPE < 3% during overground walking. The two Fitbit models produced the highest number of false positive steps during arm movements, and the two Withings devices produced the least number of false positives.

We included the ActiGraph as a scientific benchmark for the CAMs and smartphones. The ActiGraph proved to be superior to wrist-worn devices in all three protocols and to the hip-worn Fitbit Inspire during overground and intermittent walking. The ZTM algorithm that processed data from three different hip-worn smartphone models was comparable to the ActiGraph for overground walking and even superior to the ActiGraph for ADLs and intermittent walking. This held true regardless of smartphone model. We deliberately choose smartphone models from a medium to low price range to ensure generalizability of results among models. It can be assumed that the ZTM algorithm can reproduce its performance in our study with acceleration data from a majority of available smartphone models. Based on these observations, the ankle-worn Fitbit Inspire and hip-worn smartphones using the ZTM algorithm can be considered as good alternatives to the research-grade ActiGraph for measuring steps during overground walking, the ADLs tested and intermittent walking. However, this observation still needs to be reproduced in free-living conditions. Furthermore, researchers are advised to base their decisions for a specific device not merely on measurement accuracy. Especially, when longer measurement periods in free-living conditions are planned, factors like wear comfort, battery life, and usability gain importance and should be considered as well.

It is important to mention that measurement errors of all devices were considerably higher during the ADL and the intermittent walking protocol as compared to the overground walking protocol. No common thresholds of acceptable MAPEs exist for these types of protocols. However, when the threshold that is commonly used for free-living protocols (MAPE < 10%) [45,46] is applied, no device measures with acceptable accuracy. Improving devices’ step-detection algorithms for these activities may be valuable and lead to improved step estimates in free-living conditions.

### 4.5. Limitations

This study had several limitations. The study sample was rather small and homogenous. Results, therefore, may only apply to healthy young adults. Due to the low sample size, results of the intermittent walking and arm movement protocols have to be interpreted cautiously. Furthermore, the inclusion of outliers in our analysis may have influenced the results. We could not identify any specific pattern of participants, test protocol, or devices that may explain the occurrence of outliers. Future research investigating factors that produce such extreme values is needed. Other limitations arise from test protocols. First, the high standardization of the ADLs may have caused movements to deviate from movements observed in free-living conditions. Particularly, the continuous arm movements during the stacking of plates and the simulated eating behavior may have caused the measurement error to be higher than in free-living conditions. Thus, ecological validity of this test protocol has to be analyzed in future studies. Second, turns and rest periods during intermittent walking may have varied between participants. This complicated interpretation of results. Some participants turned on one foot without foot displacement, others turned by taking steps on the spot. Furthermore, we did not control the length of the rest periods between walking bouts. Thus, rest periods likely deviated from one second. Both the technique used to turn and the duration of rest periods may influence step detection. The attachment sites used in this study are also subject to some limitations. Since four devices were attached to each wrist, some wear positions were unusual and not as recommended by the manufacturer. We tried to correct for this with randomized permutation of attachment sites for each device but cannot rule out bias. Furthermore, smartphones were firmly attached to the hip or calf with a case and a strap. However, smartphones are usually carried in pockets of pants or skirts, purses, or against the breast during everyday life [47,48]. Future studies need to determine the validity of steps calculated with the ZTM algorithm at those attachment sites. Another limitation of the study is that three researchers alternatingly operated as the first assessor, attached the devices, and instructed the tests in different participants. Differences in performing these tasks may have produced bias. Lastly, one limitation of this study is that reliability of step measurement was not investigated. However, this is equally important as validity and needs to be determined for the CAMs and the ZTM algorithm under applied testing conditions.

## 5. Conclusions

This study has shown that some, but not all, activity monitors can measure steps accurately (i.e., MAPE < 3%) during overground walking. In general, hip- and ankle-worn devices clearly outperformed wrist-worn devices during overground walking, ADLs, and intermittent walking. Across devices, the type of activity protocol strongly influenced measurement accuracy, with continuous walking showing the best accuracy, followed by intermittent walking and arm movements. Wrist-worn activity monitors were particularly prone to falsely identify arm movements as steps, the extent of which was manufacturer-specific. This needs to be considered when choosing activity monitors for measuring steps during free-living PA. Data from our study can help to inform researchers’ or consumers’ decisions on which device and attachment site to choose for specific purposes.

In this study, step detection by the ZTM algorithm based on hip-worn smartphone data was comparable or even superior to step detection by the research-grade ActiGraph in all test protocols. This highlights the potential of smartphones for PA measurement. This applies all the more if validity of algorithms can be confirmed when smartphones are worn at common positions like pockets or purses.

Further studies are needed to increase understanding of activity types that presumably contribute to measurement error under free-living conditions and to be able to better quantify underestimation during intermittent, short walking bouts and overestimation during arm movements. To this end, additional test protocols need to be developed, reported in detail, and tested for ecological validity.

## Figures and Tables

**Table 1:** Specifications of activity monitors and smartphones.

Manufacturer	Device (Type)	Sensors	Price ^1^ (€)	Placement			
				Wrist	Hip	Calf	Ankle ^2^
Fitbit Inc., San Francisco, CA, USA	Inspire (CAM)	3-axis accelerometer	76	x	x		x^2^
	Ionic (CAM)	3-axis accelerometer, altimeter, gyroscope, PPG, GPS, ambient light sensor	229	x			
Garmin Ltd., Olathe, KS, USA	vivofit 4 (CAM)	Accelerometer	60	x			
	vivomove HR (CAM)	Accelerometer, barometer, PPG, ambient light sensor	163	x			
Withings, Issy-les-Mouline-aux, France	Pulse HR (CAM)	3-axis accelerometer, PPG, ambient light sensor	100	x			
	Steel HR (CAM)	3-axis accelerometer, day and night motion sensor	145	x			
Xiaomi Corp. Beijing, China	Mi Band 3 (CAM)	3-axis accelerometer, PPG	27	x			
Samsung, Seoul, South Korea	Galaxy Watch Active (CAM)	Accelerometer, barometer, gyroscope, PPG, light sensor	187	x			
ActiGraph LLC, Pensacola, FL, USA	wGT3X-BT (RGAM)	3-axis accelerometer, wear time sensor, ambient light sensor	239 ^3^		x		
Samsung, Seoul, South Korea	Galaxy S10e (smartphone)	Accelerometer, barometer, gyroscope, proximity sensor, hall sensor, geomagnetic sensor, light sensor	564		x		
Nokia Corporation, Espoo, Finland	Nokia 8 (smartphone)	Accelerometer, barometer, gyroscope, proximity sensor, e-compass, hall sensor, light sensor	250		x	x	
Sony Corporation, Tokyo, Japan	Xperia 10 (smartphone)	Accelerometer, barometer, gyroscope, proximity sensor, e-compass, hall sensor, magnetometer, step counter, significant motion detector, light sensor	285		x		

x = device placement in this study, CAM = consumer activity monitor, GPS = Global Positioning System, PPG = photoplethysmography, RGAM = research-grade activity monitor, ^1^ acquisition cost for this study, ^2^ attachment site is not recommended by manufacturer, ^3^ additional expenses are required for accompanying software.

**Table 2:** Participant characteristics.

Characteristics	*N* = 18
Male/Female (n)	7/11
Age (years)	28.8 (5.0)
Height (cm)	173.9 (10.4)
Weight (kg)	70.2 (17.1)
Dominance foot (r/l) (n)	14/4
Dominance hand (r/l) (n)	17/1

Age, height, and weight are presented as mean (standard deviation).

**Table 3:** Mean difference (step count activity monitor—actual step count) and mean average percentage error for the three activity protocols.

Activity Monitors	Overground Walking			ADLs			Intermittent Walking		
	*n*	Mean Difference (SD)	MAPE (%)	*n*	Mean Difference (SD)	MAPE (%)	*n*	Mean Difference (SD)	MAPE (%)
Fitbit Inspire	54	−12.0 (17.0)	5.3	18	108.6 (38.5)	861.2	17	7.9 (59.1)	28.8
Fitbit Ionic	54	−6.1 (32.1)	8.4	18	87.1 (37.5)	704.9	17	−9.8 (60.0)	30.8
Garmin vivofit	49	−1.2 (20.6)	2.9 *	18	45.6 (30.6)	379.7	17	−9.9 (80.2)	37.6
Garmin vivomove	54	0.3 (7.8)	1.7 *	18	59.2 (41.1)	491.9	17	−38.8 (51.1)	28.3
Withings Pulse	54	−12.0 (38.8)	5.7	17	30.5 (31.9)	239.9	17	−29.6 (59.1)	30.2
Withings Steel	54	−14.7 (48.2)	6.6	18	26.7 (28.5)	208.6	17	−41.9 (58.3)	30.3
Mi Band 3	54	−10.6 (24.1)	4.9	18	31.7 (35.3)	269.6	17	−63.8 (73.9)	47.3
Samsung Galaxy	53	−15.4 (35.5)	6.3	18	43.1 (37.2)	358.6	17	−18.8 (80.3)	39.5
Fibit Inspire (hip)	54	−12.1 (54.5)	5.8	18	−12.2 (4.1)	91.3	17	−13.3 (42.9)	19.3
Fitbit Inspire (ankle)	54	1.9 (4.2)	0.9 *	18	−4.9 (5.7)	48.2	17	46.1 (32.0)	31.2
Samsung Galaxy S10e	54	0.2 (2.0)	0.3 *	18	10.3 (6.9)	86.6	17	0.8 (41.7)	12.3
Nokia 8	54	−0.1 (2.8)	0.4 *	18	10.4 (9.3)	89.0	17	17.8 (16.6)	11.2
Sony Xperia	54	0.1 (3.0)	0.6 *	18	9.8 (7.1)	86.1	17	11.7 (24.3)	12.5
Nokia 8 (calf)	54	−95.9 (41.5)	38.2	18	9.1 (8.6)	83.8	17	−36.7 (38.7)	26.3
ActiGraph	54	−0.1 (2.4)	0.5 *	18	22.3 (7.3)	177.3	17	29.8 (19.4)	17.8

ADL = activities of daily living, MAPE = mean average percentage error, SD = standard deviation, * acceptable accuracy of MAPE < 3%.

**Figure A1:**
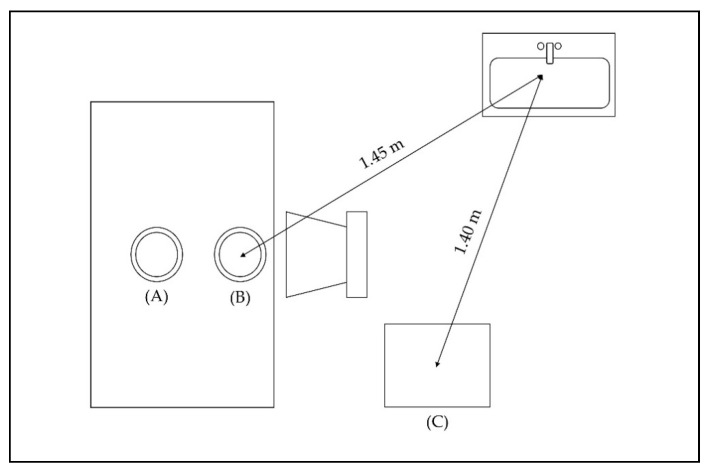
Test setup for the ADL protocol. (A) Bowl with candies, (B) stack of 10 white plates on top of one red plate, (C) starting and finishing point.

## Appendix A

Prior to the test, participants received the following instructions:

1. Stand at the starting point.
2. Turn around and walk to the hand wash basin to wash your hands with water and soap. Dry your hands with a towel.
3. Walk to the table and sit down on the chair next to the table.
4. Take five white plates and put them on the right side of the stack (one at a time), while using both hands together.
5. Repeat the same task for the other five white plates, but this time, put them on the left side of the stack.
6. Take 10 candies from the bowl (one at a time) with your right hand and then put them on the red plate.
7. Repeat the same task with your left hand.
8. Bring 10 candies (one at a time) with your right hand to your mouth (just acting like eating them) and then put them on the table next to the red plate.
9. Repeat the task with your left hand.
10. Stand up, take the red plate to the hand wash basin, wash it up, and then dry it with the towel.
11. Come back to the table, sit down again on the chair next to the table, and put the red plate back where it was before.
12. Take all five plates from the right side of the red plate (one at a time) and place them on the red plate, while using both hands together.
13. Repeat the same task for the other five white plates.
14. Stand up and walk to the finishing point.
